# A low-cost dpMIG-seq method for elucidating complex inheritance in polysomic crops: a case study in tetraploid blueberry

**DOI:** 10.1093/hr/uhae248

**Published:** 2024-09-04

**Authors:** Kyoka Nagasaka, Kazusa Nishimura, Ko Motoki, Keigo Yamagata, Soichiro Nishiyama, Hisayo Yamane, Ryutaro Tao, Ryohei Nakano, Tetsuya Nakazaki

**Affiliations:** Graduate School of Agriculture, Kyoto University, 4-2-1, Shiroyamadai, Kizugawa 619-0218, Japan; Graduate School of Agriculture, Kyoto University, 4-2-1, Shiroyamadai, Kizugawa 619-0218, Japan; Graduate School of Environmental, Life, Natural Science and Technology, Okayama University, 1-1-1, Tsushima-naka, Kita-ku, Okayama 700-8530, Japan; Graduate School of Agriculture, Kyoto University, 4-2-1, Shiroyamadai, Kizugawa 619-0218, Japan; Graduate School of Environmental, Life, Natural Science and Technology, Okayama University, 1-1-1, Tsushima-naka, Kita-ku, Okayama 700-8530, Japan; Graduate School of Agriculture, Kyoto University, 4-2-1, Shiroyamadai, Kizugawa 619-0218, Japan; Graduate School of Agriculture, Kyoto University, Kitashirakawa Oiwake-cho, Sakyo-ku, Kyoto 606-8502, Japan; Graduate School of Agriculture, Kyoto University, Kitashirakawa Oiwake-cho, Sakyo-ku, Kyoto 606-8502, Japan; Graduate School of Agriculture, Kyoto University, Kitashirakawa Oiwake-cho, Sakyo-ku, Kyoto 606-8502, Japan; Graduate School of Agriculture, Kyoto University, 4-2-1, Shiroyamadai, Kizugawa 619-0218, Japan; Graduate School of Agriculture, Kyoto University, 4-2-1, Shiroyamadai, Kizugawa 619-0218, Japan; Office of Institutional Advancement and Communications, Kyoto University, Yoshida-honmachi, Sakyo-ku, Kyoto 606-8501, Japan

## Abstract

Next-generation sequencing (NGS) library construction often requires high-quality DNA extraction, precise adjustment of DNA concentration, and restriction enzyme digestion to reduce genome complexity, which results in increased time and cost in sample preparation and processing. To address these challenges, a PCR-based method for rapid NGS library preparation, named dpMIG-seq, has been developed and proven effective for high-throughput genotyping. However, the application of dpMIG-seq has been limited to diploid and polyploid species with disomic inheritance. In this study, we obtained genome-wide single nucleotide polymorphism (SNP) markers for tetraploid blueberry to evaluate genotyping and downstream analysis outcomes. Comparison of genotyping qualities inferred across samples with different DNA concentrations and multiple bioinformatics approaches revealed high accuracy and reproducibility of dpMIG-seq-based genotyping, with Pearson's correlation coefficients between replicates in the range of 0.91 to 0.98. Furthermore, we demonstrated that dpMIG-seq enables accurate genotyping of samples with low DNA concentrations. Subsequently, we applied dpMIG-seq to a tetraploid F_1_ population to examine the inheritance probability of parental alleles. Pairing configuration analysis supported the random meiotic pairing of homologous chromosomes on a genome-wide level. On the other hand, preferential pairing was observed on chr-11, suggesting that there may be an exception to the random pairing. Genotypic data suggested quadrivalent formation within the population, although the frequency of quadrivalent formation varied by chromosome and cultivar. Collectively, the results confirmed applicability of dpMIG-seq for allele dosage genotyping and are expected to catalyze the adoption of this cost-effective and rapid genotyping technology in polyploid studies.

## Introduction

Polyploids can be divided into allopolyploid and autopolyploid on the basis of their origin. Chromosome pairing exclusively between homologous chromosomes during meiosis rather than homoeologous chromosomes results in a disomic inheritance that is often observed in allopolyploid. When one homologous chromosome has the potential to pair with all other homologous chromosomes, bivalent and multivalent chromosomes consisting of different homologous chromosome combinations can be observed, which results in a polysomic inheritance that is often observed in autopolyploid [1]. Efficient and precise genotyping is crucial for analyzing the inheritance of parental alleles within populations. Several methods for generating DNA markers have been developed, primarily involving digestions with restriction enzymes and/or polymerase chain reaction (PCR), such as random amplified polymorphic DNA, simple sequence repeats (SSR), and cleaved amplified polymorphic sequences [2–5]. These techniques have been employed to infer segregation ratios of parental alleles in diploids and polyploids [6–9], thereby confirming inheritance patterns. However, these techniques often produce a limited number of markers, and genotyping based on gel images is not well-suited for allele dosage estimation, resulting in a restricted combination of parental genotypes.

The recent development of next-generation sequencing (NGS) technology has revolutionized genotyping, enabling high-throughput detection of numerous polymorphisms. Various sequencing library construction methods with different levels of reduced genome complexity have been developed, allowing the comprehensive analysis of many samples. These methods include restriction site-associated DNA sequencing (RAD-seq) [10], genotyping by sequencing [11], double-digest RAD-seq (ddRAD-seq) [12], multiplexed inter-simple sequence repeat (ISSR) genotyping by sequencing (MIG-seq) [13, 14]. These techniques have been extensively utilized for gaining single nucleotide polymorphisms (SNPs). To facilitate genetic analysis in polyploid species, several software packages for allele dosage estimation, such as polyRAD [15] and updog package [16], have been incorporated with NGS [17–19]. Comprehensive allele dosage information across the genome is now utilized for linkage map construction and estimation of meiotic process using the software packages like MAPpoly [20], PolyOrigin [21], and polyqtlR [22] (e.g. [23–25],).

RAD-seq sequences short DNA fragments adjacent to specific restriction enzyme recognition sites, allowing flexibility in the number of detectable SNPs depending on the restriction enzymes used [10]. ddRAD-seq enhances flexibility and robustness in region recovery by utilizing two restriction enzymes [12]. However, the need for digestions with restriction enzymes limits the application of RAD-seq-based methods on samples with low-quality DNA. In contrast, MIG-seq is based on PCR using multiplexed ISSR primers, and is suitable for constructing sequencing libraries from low-quality DNA [13]. Originally developed for medium-scale studies, MIG-seq has primarily been employed for phylogenetic analysis and species discovery [26, 27]. In 2022, Nishimura *et al.* demonstrated the application of MIG-seq in quantitative trait locus analysis of wheat (*Triticum* spp.) and proved its effectiveness in genetic analysis of species with genome sizes larger than a few Gb due to the relationship between genome size and the number of SSRs [28]. However, MIG-seq is unsuitable for genetic analysis with smaller genome sizes, necessitating the development of alternative sequencing methods.

In this context, we developed degenerate oligonucleotide primer MIG-seq (dpMIG-seq), a low-cost and simple PCR-based method tailored for plants with smaller genome sizes [29]. dpMIG-seq employs ISSR PCR primers in MIG-seq [13], replacing part of the primer sequences with degenerate oligonucleotides to increase genome positions annealed by PCR primers [29]. This technique eliminates the need for DNA purification and adjustment of DNA concentrations, allowing sequencing library construction even from lysate, while maintaining flexibility in the number of detectable SNPs, thus inheriting features from both MIG-seq and RAD-seq [29]. In the original study, dpMIG-seq demonstrated its application in QTL analyses for rice (*Oryza sativa* L.), tomato (*Solanum lycopersicum* L.), and soy (*Glycine max* L.). However, its use in genetic analysis has so far been limited to diploid and polyploid species with disomic inheritance, and there has been no evaluation of the effect of the dpMIG-seq library, constructed from low-quality DNA and based on PCR, on genotyping multiple heterozygous states (i.e., allele dosage genotyping in polyploid). Since relatively high-sequencing depth is recommended for accurate dosage genotyping of polyploids (e.g. a read depth of 25 for tetraploids [16]), a cost-effective and rapid sequencing method is highly demanded. Therefore, the suitability of dpMIG-seq for polyploid samples warrants further investigation.

Blueberry (*Vaccinium* spp.) is a shrubby fruit tree, existing in diploid (2n = 2x = 24), tetraploid (2n = 4x = 48), and hexaploid (2n = 6x = 72) forms [30]. The mode of inheritance in blueberry had been controversial; segregation patterns of a specific trait or a limited number of molecular markers suggested polysomic inheritance [6, 31–33] while diversity of nucleotide sequences and subgenome-specific gene expression suggested allopolyploid origin [34]. The recent study by Mengist *et al.*, utilizing software packages for the genomic analysis of polyploids, confirmed that six tetraploid highbush blueberries (*Vaccinium corymbosum* L.) showed polysomic inheritance [25]. However, we propose to investigate the inheritance patterns of other cultivars to clarify how common their findings are across *V. corymbosum* and its hybrid species.

In this study, we first evaluated the accuracy and reproducibility of dpMIG-seq using tetraploid blueberry to confirm its applicability to polyploid samples. A comparison of genotyping results between dpMIG-seq data and resequencing data showed the effectiveness of dpMIG-seq in estimating allele dosage. Then, we applied dpMIG-seq technology to a tetraploid highbush blueberry F_1_ population and constructed an integrated linkage map to investigate inheritance patterns. The association of polysomic inheritance in tetraploid blueberry with quadrivalent formation and double reduction was demonstrated. In addition, the possibility of preferential pairing was discussed.

## Materials and methods

### Plant materials and DNA sample preparation

Tetraploid highbush blueberries ‘Spartan’ (SP) and ‘Blue Muffin’ (BM) were used as seed parent and pollen parent, respectively. In 2022, we crossed these two parental cultivars to generate an F_1_ population.

Total DNA extraction was performed from 2021 to 2022 to prepare two sets of DNA samples for different purposes: for the investigation of the optimal procedures for constructing dpMIG-seq libraries of tetraploid genomes (SAMPLE1) and for the construction of linkage maps of the parents (SAMPLE2). SAMPLE1 was prepared as follows. DNA was extracted from the leaves of BM in the autumn of 2021 and the spring of 2022 as biological replicates using a DNeasy Plant Mini Kit (Qiagen, Hilden, Germany). In the autumn of 2022, DNA was also extracted from the leaves of BM using a NucleoBond HMW DNA kit (Macherey-Nagel, Düren, Germany). A portion of the DNA sample was repeatedly diluted with sterilized water to prepare eight DNA samples that differed only in concentration. DNA concentrations of the diluted samples were measured using a Qubit™ dsDNA HS Assay Kit (Thermo Fisher Scientific, MA, USA). SAMPLE2 was prepared as follows. DNA was extracted from the leaves of parental cultivars and 256 individuals in the F_1_ population. The DNeasy Plant Mini Kit was used for the parental cultivars, whereas a simplified method using the AP1 buffer from the DNeasy Plant Mini Kit (Qiagen), described by Mizuno *et al.* (2020) [35], was applied to the F_1_ population.

### Sequencing and allele dosage estimation

dpMIG-seq libraries were constructed for SAMPLE1 and SAMPLE2 [29]. Initially, multiplex PCR was performed using Multiplex PCR Assay Kit ver. 2 (TAKARA Bio Co. Ltd., Kusatsu, Japan) and primers (Table S1) [29]. The PCR conditions involved an initial denaturation step at 94°C for 1 minute, followed by 25 cycles of denaturation at 94°C for 30 seconds, annealing at 38°C for 1 minutes, extension at 72°C for 1 minute, and a final extension at 72°C for 10 minutes. The resulting PCR product was diluted 50-fold, which employed indexing primers [28] and PrimeSTAR GXL DNA Polymerase (TAKARA Bio Co. Ltd.). The second PCR conditions included an initial denaturation at 98°C for 30 seconds, followed by 20 cycles of denaturation at 98°C for 10 seconds, annealing at 54°C for 15 seconds, extension at 68°C for 30 seconds, and a final extension at 72°C for 10 minutes. Subsequently, the second PCR products were pooled, purified using AMPure XP (Beckman Coulter, Inc., CA, USA), and subjected to reconditioning PCR with conditions including 98°C for 40 seconds, 54°C for 15 seconds, 68°C for 30 seconds, and a final extension at 72°C for 10 minutes. Following purification using AMPure XP, suitable fragments for sequencing were selected using SPRIselect (Beckman Coulter, Inc.). The obtained dpMIG-seq libraries were sequenced on the Illumina HiSeq X platform using 151-cycle paired-end runs. Samples with different DNA concentrations in SAMPLE1 were sequenced twice using separate dpMIG-seq library preparations as technical replicates. For BM gDNA extracted using the NucleoBond HMW DNA kit, whole genome resequencing was carried out on the NovaSeq platform and 150 bp paired-end reads were obtained.

Using fastp (version 0.19.5) [36], raw reads were filtered with default settings except reads with a base-quality Phred score of less than 20 and a read length of less than 35, which were discarded. At the same time, for reads from the dpMIG-seq libraries, 17 base primer sequences in the first PCR of dpMIG-seq [29] were trimmed. Clean reads were aligned to the 12 largest chromosomes of each homologous set from the ‘Draper’ reference genome ^[34]^ using BWA-MEM (version 0.7.17-r1188) [37]. Downsampling was performed for the aligned reads in SAMPLE1 using the shuf command. For the technical replicates, 1.5 million aligned reads were extracted randomly, whereas for the biological replicates, 1, 2, 3, 4, 5, 6, 7, and 8 million aligned reads were extracted randomly and separately. SNP calling was performed using the mpileup command in SAMtools (version 1.9) [38] and the mpileup2snp command in VarScan (version 2.4.4–0) [39], and alignments with mapping quality less than 20 were discarded. The initial vcf file was exploited for the evaluation of dpMIG-seq-based genotyping as follows.

### Reproducibility and accuracy of genotyping by dpMIG-seq

By using the above procedures, ‘VCF1’ that contains SNP information for all of the DNA samples from SAMPLE1 and SAMPLE2 was obtained. VCF1 was then filtered using VCFtools (version 0.1.16) [40] with the following criteria: (i) minimum depth of 10, 20, 30, or 40 (option—minDP 10, 20, 30, or 40) for dpMIG-seq data and 75 (option—minDP 75) for resequencing data; (ii) maximum depth of 5000 (option—maxDP 5000) for dpMIG-seq data and 300 (option—maxDP 300) for resequencing data; (iii) maximum missing data of 0.9 (option—max-missing 0.1); (iv) minor allele frequency of 0.05 (option—maf 0.05); and (v) only biallelic loci. Subsequently, depths of reference allele-supporting bases and alternative allele-supporting bases were extracted from VCF1 per locus and per sample. Allele dosage was estimated using the updog package (version 2.1.3) [16] with default settings except for the ploidy level, in which loci with less than 0.05 posterior proportion of mis-genotyped individuals were retained.

VCF1 and allele dosage from VCF1 were used to compare genotyping results between technical and biological replicates and between dpMIG-seq and resequencing data. We counted the number of SNPs detected and then performed a set operation to count the number of SNPs belonging to union and intersection between technical and biological replicates. As regards intersection of SNPs, alternative allele frequencies were calculated as alternative allele-supporting bases/(reference allele-supporting bases + alternative allele-supporting bases) per locus and per sample, and correlation in the alternative allele frequencies was investigated on the basis of Pearson’s correlation coefficients. In addition, SNPs whose genotyping results were concordant between the technical/biological replicates but were discordant between the sequencing methods were examined to determine how the genotyping results differed.

### Linkage map construction

In the same way as VCF1, ‘VCF2’, which contains only SNP information for the parental cultivars and the F_1_ population from SAMPLE2, was obtained. VCF2 was then filtered using VCFtools with the following criteria: (i) minimum depth of 20 (option—minDP 20); (ii) maximum depth of 5000 (option—maxDP 5000); (iii) maximum missing data of 0.25 (option—max-missing 0.75); (iv) minor allele frequency of 0.05 (option—maf 0.05); (v) only biallelic loci; and (vi) no monomorphism. Subsequently, in the same way as VCF1, depths of reference allele-supporting bases and alternative allele-supporting bases were extracted from VCF2 separately, per locus and per sample. Allele dosage was estimated using the updog package with default settings except for the ploidy level, in which loci with posterior proportion of individuals mis-genotyped less than 0.05 were retained.

The integrated linkage map was constructed using MAPpoly (version 0.4.1) [20]. Before starting the map construction, SNP markers in VCF2 were subjected to several additional filtering steps following the tutorial written by the authors of MAPpoly. Markers with missing fractions across individuals higher than 0.1 and individuals with missing fractions across markers higher than 0.1 were discarded. In addition, using the expected segregation ratios in a hybrid population considering Mendelian inheritance when homologous chromosomes form bivalents with random combinations, distorted markers were removed by performing a χ^2^ test with Bonferroni correction assuming an alpha level of 0.1. Finally, markers with identical allele dosage information for all individuals were eliminated.

Pairwise recombination fractions were calculated to cluster markers into 12 linkage groups (LGs) using the function group_mappoly. The LOD score threshold for linkage phase configurations was set to 2. Chromosomes to which markers were aligned were compared with LGs to which the markers belonged to determine the chromosomes representing the LGs, leaving only matched markers. Retained markers were then ordered within each LG on the basis of the ‘Draper’ reference genome ^[34]^. Multipoint analysis in ordered marker sets was performed using the function est_rf_hmm_sequential with the following parameters slightly modified from Cappai *et al.* (2020) [41]: start.set = 20; thres.twopt = 10; thres.hmm = 10; extend.tail = 200; info.tail = TRUE; sub.map.size.diff.limit = 10; phase.number.limit = 20; reestimate.single.ph.configuration = TRUE; tol = 10e-3; tol.final = 10e-4. A single marker at the beginning of LG4, leading to map tension, was excluded. To lower the inflation of a genetic map attributed to genotyping errors, the initial map was re-estimated considering a global genotyping error of 0.1. The final linkage map was visualized by LinkageMapView [42]. Meiotic recombination rate was estimated using MareyMap (version 1.3.7) [43], following the method proposed by Mengist *et al.* (2023) [25]: loess-based method, setting Span to 0.4.

### Haplotype reconstruction and recombination point detection

Three R packages, MAPpoly, PolyOriginR (version 0.0.3) [21], and polyqtlR (version 0.0.9) [22], were used for the haplotype reconstruction of parental cultivars. The final genetic map constructed above was exploited to perform the following analysis.

Haplotype reconstruction by MAPpoly was performed by calculating the conditional probability of all possible 36 genotypes, or eight haplotype combinations per locus and per individual, and then determining the homolog probabilities, or the probability of the locus composed of the specific combination of eight haplotypes. The functions calc_genoprob_error and calc_homologprob implemented in MAPpoly were used, and the global error of 0.1 was assumed.

MAPpoly assumes only random bivalent formation, whereas PolyOriginR and polyqtlR support quadrivalent formation. Haplotype reconstruction with PolyOriginR was performed using the function PolyOriginR, assuming two chromosome pairing conditions: bivalent formation only (chrpairing = 22), and bivalent and quadrivalent formation (chrpairing = 44). Additionally, error priors of 0.01, 0.05, and 0.1 were considered (epsilon = 0.01, 0.05, or 0.1). As regards polyqtlR, IBD probabilities were estimated using the function estimate_IBD, similarly assuming bivalent formation only (bivalent_decoding = TRUE), and bivalent and quadrivalent formation (bivalent_decoding = FALSE). Additionally, error priors of 0.01, 0.05, and 0.1 were considered (error = 0.01, 0.05, or 0.1). The inherited haplotypes of individuals were examined using the function visualiseHaplo.

Recombination breakpoints were detected across the genome using the function count_recombination in polyqtlR. There might be scenarios where more than one plausible pairing configuration exists. Therefore, the number of recombination breakpoints between chromosomes that paired with a probability of 0.5 or higher was counted for each chromosome to exclude inconclusive pairing configurations. Note that the function count_recombination was developed in the context of bivalent pairing, meaning that only individuals predicted to have come from meiosis with only bivalents were used in the calculation [22].

### Statistical tests for preferential chromosomal pairing

The three R packages, MAPpoly, PolyOriginR, and polyqtlR, were also used to estimate chromosome pairing during meiosis. MAPpoly computes the posterior probability of the pairing configurations on a locus scale, whereas PolyOriginR and polyqtlR deal with posterior probability of the pairing configurations on a chromosome scale.

For MAPpoly, the χ^2^ test was performed for all the loci using the function calc_prefpair_profiles. The function p.adjust implemented in R was used to adjust p values for multiple comparisons with the Benjamini & Hochberg method [44] (FDR < 0.05).

For PolyOriginR and polyqtlR, the functions PolyOriginR and meiosis_report were used to calculate the proportion of specific homologous chromosome pairs, including bivalents and quadrivalents, observed in the mapping population. Thresholds for the probability of pairing configurations were set to 0.8, 0.5, or 0.3. As autotetraploids with random chromosome pairing have a one-in-three chance of forming a bivalent in a particular chromosome combination, the χ^2^ test was performed on the estimated proportion of three chromosome combinations to examine deviations from one-third (*P* < 0.05).

### Detection of double reduction

PolyOriginR and polyqtlR were used to calculate the double reduction rate. As discussed earlier, the probabilities of an individual inheriting particular alleles were calculated using the functions PolyOriginR and estimate_IBD implemented in PolyOriginR and polyqtlR, respectively. For a given marker, the probabilities of inheriting alleles from sister chromatids were summed up and then divided by the number of individuals analyzed. Double reduction rates per marker were then averaged for 1 Mb windows.

## Results

### Reproducibility and accuracy of genotyping results based on dpMIG-seq

**Figure 1:**
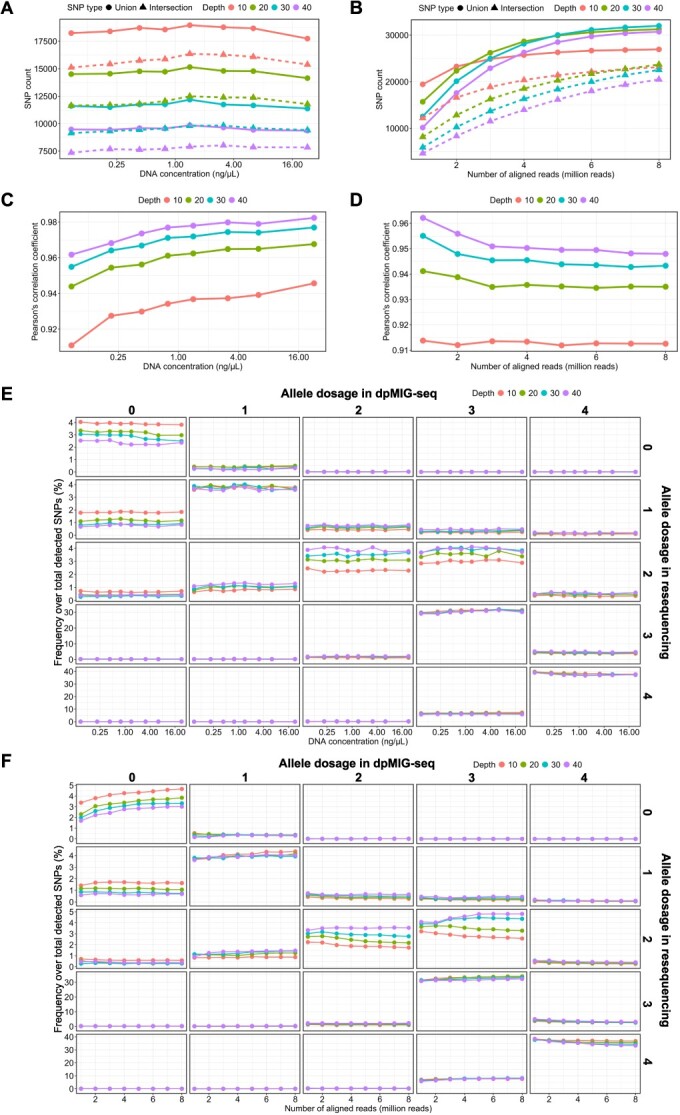
**Evaluation of genotyping reproducibility and accuracy by dpMIG-seq.** The number of SNPs depending on (A) DNA concentration and (B) the number of aligned reads. Union and Intersection represent the number of SNPs detected in either and both of the replicates, respectively. Pearson’s correlation coefficient of alternative allele frequencies depending on (C) DNA concentration and (D) the number of aligned reads. The percentage of SNPs with discordant genotyping results over total detected SNPs depending on (E) DNA concentration and (F) the number of aligned reads, respectively. The numbers presented at the top of each column and on the left side of each row represent the reference allele dosage from dpMIG-seq data and resequencing data, respectively. The diagonal sections illustrate changes in the percentage of SNPs with concordant genotyping results, while the remaining sections indicate those with discordant genotyping results. For (A), (C), and (E), the x-axis was log2-transformed.

In VCF1, the total numbers of SNPs detected with minimum depths of 10, 20, 30, and 40 were 122 334, 87 761, 69 701, and 57 749 SNPs, respectively. Among these, 27 788, 32 657, 34 045, and 32 833 SNPs were assigned allele dosage with less than 0.05 posterior proportion of mis-genotyped individuals at minimum depths of 10, 20, 30, and 40, respectively. Technical replicates were employed to investigate the effect of DNA concentrations on genotyping results, while biological replicates were utilized to assess the effect of the number of aligned reads on genotyping results. For technical replicates, the total number of SNPs detected in union and intersection between technical replicates increased as the minimum depth decreased, whereas the sample diluted 128-fold yielded almost the same number of SNPs as the undiluted sample (Fig. 1A). For biological replicates, the number of SNPs detected in union and intersection between biological replicates increased whereas the SNP increment rate decreased as the number of aligned reads increased (Fig. 1B). The percentages of SNPs in intersection relative to those in union ranged from 78% to 87% for technical replicates and from 46% to 86% for biological replicates. The larger the number of aligned reads, the greater the likelihood that SNPs at the same locus will be detected across biological replicates. For example, for 1 million and 8 million alignments, the percentage of SNPs in intersection at the minimum depth of 10 was 63% and 86%, respectively. In order to apply dpMIG-seq to polyploids, the reproducibility of the allele dosage must be guaranteed. First, we calculated Pearson’s correlation coefficients between technical/biological replicates to determine the stability of alternative allele frequencies. Pearson’s correlation coefficients between technical replicates ranged from 0.91 to 0.98. Although slightly better results were obtained at higher DNA concentrations, genome-wide alternative allele frequencies showed a high degree of similarity between technical replicates, surprisingly, even at very low DNA concentrations (Fig. 1C). For biological replicates, Pearson’s correlation coefficients ranged from 0.91 to 0.96 and tended to be slightly lower as the number of aligned reads increased (Fig. 1D). Overall, a larger depth value yielded a slightly higher Pearson’s correlation coefficient. Subsequently, allele dosage estimated from the allele frequencies was compared between replicates (Fig. S1). For technical replicates, the number of SNPs whose genotyping results matched remained almost unchanged notwithstanding changes in DNA concentration, consistent with the allele frequency results (Fig. S1A, represented by triangles). As for biological replicates, the number of SNPs with concordant genotyping results increased as the number of aligned reads increased, but the SNP increment rate showed a downward trend (Fig. S1B, represented by triangles). The percentage of SNPs with concordant genotyping results among SNPs in intersection ranged from 89% to 95% between technical replicates and from 95% to 97% between biological replicates. Overall, a small minimum depth resulted in a large number of SNPs with concordant genotyping results.

The accuracy of genotyping results obtained from the dpMIG-seq data was validated by comparing it with that of genotyping results obtained from the resequencing data, which are supposed to represent more accurate genotypes. The number of SNPs with concordant genotyping results between the dpMIG-seq data and the resequencing data was independent of DNA concentration but increased as the number of aligned reads increased (Fig. S1, represented by squares). On the other hand, the percentage of SNPs with concordant genotyping results between the dpMIG-seq data and the resequencing data was relatively low, ranging from 71% to 75% among the dpMIG-seq data with different DNA concentration, and from 72% to 79% among the dpMIG-seq data with different numbers of aligned reads. In addition, we investigated which genotypes tended to be assigned an incorrect allele dosage. The most frequent discordant type was when dpMIG-seq data indicated a reference allele dosage of 3, but resequencing data supported a reference allele dosage of 4. This discordant genotyping result accounted for from 6% to 7% and from 6% to 8% of total detected SNPs with different DNA concentrations (Fig. 1E) and different numbers of aligned reads (Fig. 1F), respectively. This discordant result comprised from 14% to 17% and from 14% to 18% of SNPs with a reference allele dosage of 3 as inferred by dpMIG-seq, for samples with different DNA concentrations and different numbers of aligned reads, respectively. Meanwhile, the most challenging aspect in genotyping appeared to be determining a reference allele dosage of 2. The percentage of SNPs inferred to have a reference allele dosage of 2 by both dpMIG-seq and resequencing data was from 2% to 4% of total detected SNPs. Given that the percentage of total SNPs with a reference allele dosage of 2 supported by resequencing data was from 7% to 10% and from 6% to 11% for different DNA concentrations (Fig. 1E) and different numbers of aligned reads (Fig. 1F), respectively, over half of the SNPs with an allele dosage of 2 could not be accurately genotyped. Overall, a non-negligible proportion of SNPs was suggested to be mis-genotyped.

### Linkage map construction and reliable SNP selection

**Figure 2:**
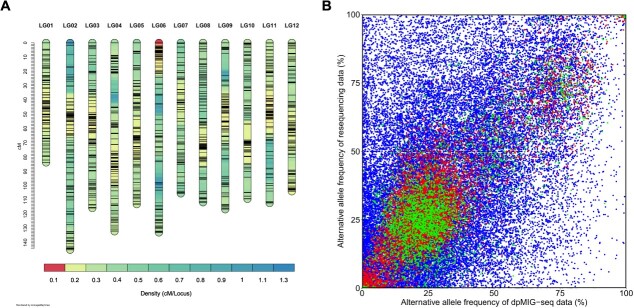
**Overview of integrated linkage map and allele frequencies of SNP markers.** (A) Density map of the 12 linkage groups. Marker density is indicated by color, and marker positions are indicated by black lines. (B) Comparison of allele frequencies between dpMIG-seq data and resequencing data when the minimum depth is 20. Green points represent allele frequencies of SNPs used for linkage map construction. Among the remaining SNPs, those with genotyping results that are concordant between dpMIG-seq data and sequencing data are represented by red points, whereas those with discordant genotyping results are represented by blue points.

The above results indicate that allele dosage estimation with dpMIG-seq is difficult in terms of accuracy. Therefore, before the estimated allele dosage is used for downstream analysis, it is necessary to exclude loci with a high proportion of mis-genotyped individuals to ensure credibility of the results. In the case of analyses of F_1_ populations and other segregating populations, allelic segregation patterns in offspring can be used to validate genotyping results, allowing for further selection of SNPs. A total of 256 individuals were genotyped and 19 695 SNPs were obtained from VCF2. Among them, four individuals and 11 191 SNPs were discarded after additional filtering. Finally, an integrated linkage map with a total map length of 1380.89 cM was created, containing 6000 markers (Table 1). The length of each LG ranged from 83.7 cM to 144.98 cM. The average map density was 4.4 markers/cM and the markers were distributed throughout the 12 LG (Fig. 2A). Additionally, marey maps depicting genetic distance plotted against physical position and transition of recombination rate across genome was compared to estimate the approximate position of the centromeres (Fig. S2).

**Table 1:** Summarized data of the integrated linkage map

LG	Map length (cM)	Markers/ cM	Simplex	Double-simplex	Multiplex	Total	Max gap (cM)
1	83.7	5.56	370	29	66	465	4.4
2	144.98	3.43	375	42	80	497	8.52
3	115.6	4.57	406	35	87	528	6.18
4	132.13	4.47	447	69	75	591	13.36
5	112.87	4.88	430	17	104	551	8.27
6	132.89	3.94	408	38	77	523	14.45
7	105.41	3.72	289	39	64	392	5.67
8	111.63	3.91	321	42	74	437	7.92
9	116.51	4.09	347	32	98	477	15.83
10	109.2	4.32	376	14	82	472	10.74
11	112.1	4.74	423	18	90	531	7.34
12	103.87	5.16	432	46	58	536	8.67
Total	1380.89	4.4	4624	421	955	6000	9.28

LG means linkage groups. Simplex, double-simplex, and multiplex represent the numbers of simplex markers, double-simplex markers, and other markers included in the map, respectively.

To confirm the reliability of the 6000 selected SNPs for linkage map construction, the alternative allele frequencies of VCF1 were visualized (Fig. 2B). If two sequencing data representing the genome of the same individual gives the same genotyping results, the scatter plot showing the correspondence of their allele frequencies should lie on line y = x. Points of allele frequencies in Fig. 2B were distributed throughout the figure and did not lie on a straight line, suggesting that a large number of genotype mismatches existed between the dpMIG-seq data and the resequencing data. In contrast, in the 6000 selected SNPs, the allele frequencies were relatively concordant between the dpMIG-seq data and the resequencing data (Fig. 2B, represented by green points). Collectively, the filtering steps performed prior to the linkage map construction eliminated problematic SNPs, contributing to the creation of a high-quality map.

### Preferential pairing

**Figure 3:**
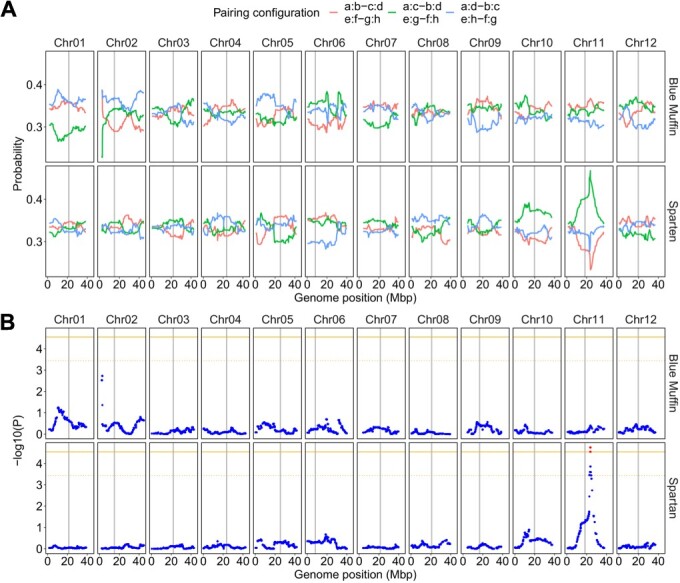
**Probability profiles across 12 linkage groups of ‘Blue Muffin’ and ‘Spartan’ for three pairing configurations.** (A) Four homologous chromosomes are represented by a–d and e–h for ‘Blue Muffin’ and ‘Spartan’, respectively. Paired chromosomes are indicated by ‘:’. For example, a:b shows that homologous chromosomes a and b form a bivalent. When homologous chromosomes pair randomly, the probability of each pairing configuration is expected to be 1/3. (B) χ2 test to examine the possibility of preferential pairing. Red points represent significant markers (FDR < 0.05). Orange solid and dotted lines represent genome-wide significance levels at 5% and 10%, respectively. Gray vertical lines represent genomic regions with the lowest recombination rates.

Fig. 3 shows the probability of each pairing configuration in relation to the position computed with MAPpoly. Under the random meiotic pairing in tetraploid, a homologous chromosome is expected to have a one-in-three chance of pairing with each of the other homologous chromosomes. In addition, when two chromosomes are paired, the remaining two chromosomes are similarly thought to form a bivalent, so three combinations of bivalents exist, namely, ‘a:b–c:d’, ‘a:c–b:d’, and ‘a:d–b:c’, where a, b, c, and d represent each of the four homologous chromosomes. Two tetraploid blueberry cultivars, SP and BM, showed an even probability of pairing configurations for most chromosomes (Fig. 3A). The only exception was chromosome 11 (chr-11) of SP, where the ‘e:g–f:h’ combination was observed at a significantly higher probability than the other combinations (FDR < 0.05) (Fig. 3B). Tables 2 and 3 show the frequencies of the three combinations of bivalents, assuming bivalent and quadrivalent formation, an error prior of 0.05, and a probability threshold for 0.5, as estimated by PolyOriginR and polyqtlR, respectively. Similarly to the results of the mappoly package, three combinations of bivalents were evenly observed for most chromosomes of both cultivars. On the other hand, some chromosomes, such as chr-2 of BM and chr-1, chr-10, and chr-11 of SP, exhibited significantly higher frequencies of certain combinations (*P* < 0.05). The frequencies of the three combinations of bivalents slightly changed by modifying the conditions for analysis, i.e., assuming only bivalent formation, the error prior of 0.01/0.1, and/or the probability threshold of 0.8/0.3 (Tables S2–S13). We found that the p values for chr-11 of SP ranged from 0.0010 to 0.0141 with PolyOriginR (Tables 2, S2–S7) and from 0.0016 to 0.0528 with polyqtlR (Tables 3, S8–S13). With regards to the remaining three chromosomes, the p values for chr-2 of BM ranged from 0.0010 to 0.0337 with PolyOriginR (Tables 2, S2–S7) and from 0.0003 to 0.0037 with polyqtlR (Tables 3, S8–S13), that for chr-1 of SP ranged from 5.8 × 10^−6^ to 0.2797 with PolyOriginR and from 0.0012 to 0.4034 with polyqtlR, and that for chr-10 of SP ranged from 0.0002 to 0.0544 with PolyOriginR and from 0.0010 to 0.0427 with polyqtlR. Owing to the large variation of p values and the need to adjust p value because we tested a total of 12 chromosomes independently, chr-1 and chr-10 of SP were not considered sufficient evidence of preferential pairing.

**Table 2:** Observed frequencies of bivalents and quadrivalents estimated by the PolyOriginR package when the error prior is 0.05 and the probability threshold is 0.5

		Blue Muffin						Spartan					
chromosome	probability	a:b-c:d	a:c-b:d	a:d-b:c	a:b:c:d	*χ* ^2^	p value	e:f-g:h	e:g-f:h	e:h-f:g	e:f:g:h	*χ* ^2^	p value
1	0.5	43	25	38	47	4.887	0.0869	57	50	27	19	11.030	0.0040
2	0.5	55	56	81	19	6.781	0.0337	66	47	62	36	3.440	0.1791
3	0.5	61	59	58	24	0.079	0.9614	63	57	66	16	0.677	0.7127
4	0.5	51	59	54	48	0.598	0.7417	60	52	57	43	0.580	0.7483
5	0.5	61	57	74	13	2.469	0.2910	62	48	52	43	1.926	0.3818
6	0.5	42	51	41	47	1.358	0.5071	49	51	42	39	0.944	0.6239
7	0.5	43	48	42	27	0.466	0.7921	42	52	42	24	1.471	0.4794
8	0.5	63	58	58	24	0.279	0.8696	49	56	52	46	0.471	0.7900
9	0.5	58	53	60	24	0.456	0.7961	44	43	61	47	4.149	0.1256
10	0.5	60	61	55	24	0.352	0.8385	45	74	49	32	8.821	0.0121
11	0.5	62	59	52	25	0.913	0.6334	36	71	47	44	12.481	0.0019
12	0.5	54	63	50	38	1.593	0.4509	54	56	61	34	0.456	0.7961

Probability denotes the threshold for plausible pairing configurations. Paired chromosomes are indicated by ‘:’. For example, a:b shows that homologous chromosomes a and b form a bivalent. χ^2^ test was performed to test for deviation from 1/3 for the observed frequencies of bivalents.

**Table 3:** Observed frequencies of bivalents and quadrivalents estimated by the polyqtlR package when the error prior is 0.05 and the probability threshold is 0.5

		Blue Muffin						Spartan					
chromosome	probability	a:b-c:d	a:c-b:d	a:d-b:c	a:b:c:d	*χ* ^2^	p value	e:f-g:h	e:g-f:h	e:h-f:g	e:f:g:h	*χ* ^2^	p value
1	0.5	80	54	80	31	6.318	0.0425	85	72	63	25	3.336	0.1886
2	0.5	67	66	109	7	14.934	0.0006	95	79	67	8	4.913	0.0857
3	0.5	79	84	81	5	0.156	0.9251	84	71	86	8	1.651	0.4379
4	0.5	82	78	77	13	0.177	0.9152	79	79	74	18	0.216	0.8978
5	0.5	76	67	101	5	7.631	0.0220	81	71	84	13	1.178	0.5549
6	0.5	60	87	83	16	5.539	0.0627	82	84	62	18	3.895	0.1426
7	0.5	73	74	74	15	0.009	0.9955	73	84	68	11	1.787	0.4093
8	0.5	84	74	80	8	0.639	0.7266	64	73	88	21	3.920	0.1409
9	0.5	78	77	85	5	0.475	0.7886	68	71	80	26	1.068	0.5861
10	0.5	79	75	75	14	0.140	0.9325	68	99	63	12	9.922	0.0070
11	0.5	84	74	72	10	1.078	0.5833	59	91	69	21	7.342	0.0254
12	0.5	73	88	62	23	4.583	0.1011	75	77	83	11	0.443	0.8015

Probability denotes the threshold for plausible pairing configurations. Paired chromosomes are indicated by ‘:’. For example, a:b shows that homologous chromosomes a and b form a bivalent. χ^2^ test was performed to test for deviation from 1/3 for the observed frequencies of bivalents.

### Quadrivalent formation and double reduction rate

**Figure 4:**
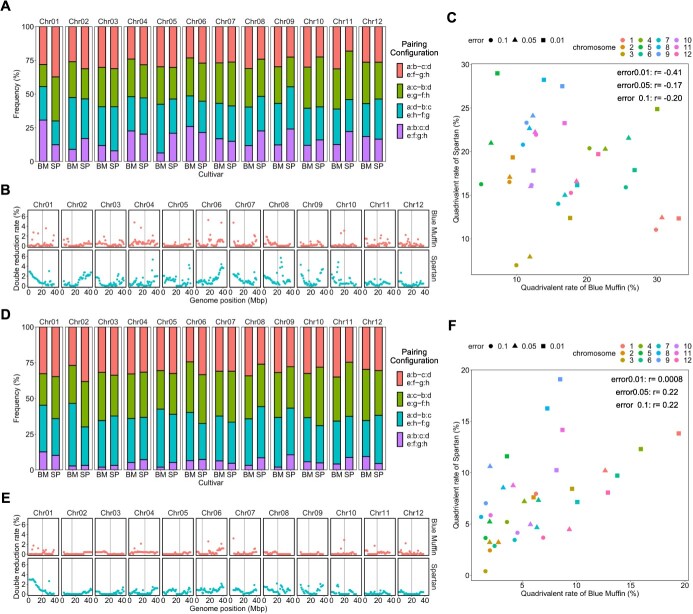
**Quadrivalent formation in ‘Blue Muffin’ and ‘Spartan’.** Results estimated from the PolyOriginR and polyqtlR packages are shown in (A)–(C) and (D)–(F), respectively. (A), (B), (D), and (E) are results obtained when the error prior is 0.05. (A), (D) Proportion of bivalents and quadrivalents when the probability threshold is 0.5. Paired chromosomes are indicated by ‘:’. For example, a:b shows that homologous chromosomes a and b form a bivalent. BM stands for ‘Blue Muffin’ and SP, for ‘Spartan’. (B), (E) Double reduction rate across 12 linkage groups. Red and blue points represent ‘Blue Muffin’ and ‘Spartan’, respectively. Gray vertical lines represent genomic regions with the lowest recombination rates. (C), (F) Correlation of the quadrivalent proportion of ‘Blue Muffin’ with that of ‘Spartan’. Each color corresponds to a different chromosome. Points shaped as square, triangle, and circle represent results when the error prior is 0.01, 0.05, and 0.1, respectively. Numbers in the figure represent Pearson's correlation coefficients, which are not significant at the 5% level.

Quadrivalents were suggested to be formed for all of the 12 chromosomes. Its proportion across the mapping population varied by chromosome and parental cultivar. Figures 4A and 4D show the proportion of quadrivalents per chromosome and parental cultivar (represented by purple areas); the percentage ranged from 6% to 31% with PolyOriginR and 2% to 13% with polyqtlR, respectively. As in the subsection ‘Preferential pairing’, the proportion of quadrivalents was slightly changed by modifying the conditions for analysis. The range of the proportion expanded from 3% to 45% with PolyOriginR (Figs. S3A, S4A, and Tables S2–S4) and from 0% to 23% with polyqtlR (Figs. S3C, S4C, and Tables S8–S10).

Accompanied by quadrivalent formation, double reduction was suggested to occur (Figs. 4B, 4E). The double reduction rate was higher at telomeres than at expected positions of centromeres. Overall, chromosomes with a high frequency of quadrivalent chromosome formation had a high double reduction rate. The results obtained with other analytical conditions are shown in Figs. S3B, S3D, S4B, and S4D.

## Discussion

### Optimization of dpMIG-seq for autotetraploid

The sequencing library construction method used in this study, named dpMIG-seq, requires two rounds of PCR steps for amplification and indexing [29]. The results showed that genotyping of low-concentration DNA samples is possible, even in tetraploids, through the PCR steps. Surprisingly, the reproducibility and accuracy of the genotyping results for the DNA sample with the lowest concentration in this study, 0.085 ng/μL, are comparable to those of the DNA sample with the highest concentration of 22.9 ng/μL. It should be noted that a DNA concentration of 4 ng/μL or higher is recommended for tetraploids when available because DNA samples with higher concentrations had slightly higher reproducibility as evidenced by Pearson’s correlation coefficients (Fig. 1C).

The reproducibility of detected loci by dpMIG-seq was guaranteed by increasing the number of aligned reads owing to the limitation of the total number of amplifiable loci across genome (Fig. 1B). Nishimura *et al.* (2024) conceived the idea of changing part of the primer sequences for the first PCR to degenerate oligonucleotides to increase the genomic regions that the primers can anneal. In tetraploid wheat (*Triticum turgidum* L.), the number of SNPs obtained by dpMIG-seq increased in proportion to the data volume when the data volume was studied up to approximately 5.5 Gb [29]. Similarly, in tetraploid blueberry, the number of SNPs increased proportionally with the data volume until exceeding 4 million reads. However, amplifiable loci may saturate with around 10 million reads in blueberry due to its smaller genome size compared to tetraploid wheat. One solution to increase detectable SNPs is to use primers with degenerate nucleotides at different positions [29]. To maximize cost-effectiveness, we recommend less than 5 million aligned reads or approximately 1 Gb of raw data when using one primer set for the first PCR, and if more SNPs are needed for analyses, we recommend trying other primers with degenerate nucleotides at different positions and merging the sequencing data. Given that the number of bases mapped with a depth of 10 or greater was relatively stable across species with varying genome sizes [29]^,^ the above data amount is also recommended for other tetraploid species initially. For species with higher ploidy, a slightly larger data amount is deduced to increase the number of SNPs with enough depth.

Because of the required PCR step, dpMIG-seq may skew the allele frequencies of the SNPs compared with methods without PCR, affecting genotyping accuracy particularly when estimating allele dosage in polyploids. Suyama and Matsuki (2015) proposed five possible artifacts, one of which was biased read depth owing to PCR error [13]. We observed that 21% to 29% of the allele dosages estimated from the dpMIG-seq data were not concordant with those estimated from the resequencing data (Fig. S1), and partially attributed this to the distortion of allele frequencies. SNPs obtained from the dpMIG-seq data were deduced to have a greater proportion of misclassified allele dosage than that from the resequencing data and affect the results of the genetic analysis. We addressed this problem by selecting markers on the basis of segregation ratios prior to the linkage map construction following the MAPpoly tutorial, and succeeded in gaining clean markers and constructing a linkage map that is collinear with the ‘Draper’ genome assembly [34] (Fig. S2) and compatible with previous research in map length (Fig. 2A, Table 1) [41, 45]. Thus, dpMIG-seq was confirmed to be applicable to a polyploid segregating population like an F_1_ population for the first time in this study.

By summing up the necessary items described in the Materials and Methods, the estimated cost per sample, from DNA extraction to library construction, was approximately one US dollar, which is about two-thirds of ddRAD-seq (Table S14). Moreover, the estimated SNP count from 5 million aligned reads of dpMIG-seq exceeded that of ddRAD-seq (Supplementary Materials and Methods; Table S14), indicating that dpMIG-seq offers superior genotyping efficiency superior to ddRAD-seq. Additionally, dpMIG-seq demonstrated higher genotyping efficiency than MIG-seq for blueberry, given its genome size of approximately 600 Mbp/haploid genome [46], corroborating findings from Nishimura *et al.* (2022) [28]. Like blueberry, dpMIG-seq is recommended for species with genome sizes under a few Gb. This cost-effective and rapid genotyping method holds promise for advancing genomic analysis in polyploids.

### Differences in inheritance patterns between chromosomes and cultivars

Statistical analysis of the mapping population derived from a cross between SP and BM showed that most homologous chromosomes formed bivalents in random combinations, which is consistent with the latest study [25]. Nevertheless, our results suggested the possibility of preferential pairing on chr-11 of SP, as consistently indicated by the three R packages. Although Mengist *et al.* (2023) considered insufficient evidence, there were some indications of deviations from random pairing for chr-11 of ‘Reveille’ [25]. Further study is needed to determine the prevalence of preferential pairing on chr-11 in blueberry cultivars and the extent to which preferential pairing affects the segregation of preferential alleles. Regarding preferential pairing for chr- 2 of BM, PolyOriginR and polyqtlR detected an over-abundance of ‘a:d–b:c’ combination (Tables 2, 3), whereas MAPpoly suggested an under-abundance of ‘a:c–b:d’ combination at the start of the chromosome (Fig. 3). Although preferred chromosome combinations are often over-represented (e.g. [23]), our results potentially indicated that preferential pairing was induced by decreased affinity of chromosome pairing. On the other hand, the discrepancies, with both decreased and increased affinity of chromosome pairing on the same chromosome, could stem from the same phenomenon and may be attributed to the differences in algorithms between packages. As only a part of the selections/cultivars examined so far showed the possibility of preferential pairing, investigating the mechanism underlying the difference in chromosome configuration frequencies between selections/cultivars may yield exciting findings.

Polyploids that exhibit polysomic inheritance may form multivalents, and the formation of multivalents has a significant impact on allelic segregation owing to double reduction. BM and SP were inferred to have different proportions of quadrivalent formation depending on cultivar and chromosome (Figs. 4, S3, S4), which was compatible to the results from Mengist *et al.* (2023) [25]. In sweetpotato, the rate of multivalent formation was positively correlated with the length of the linkage map [23]. However, we did not find any significant correlations in quadrivalent proportion per chromosome between BM and SP (Figs. 4C, 4F), suggesting that homologous chromosomes that have favorable or unfavorable conditions to form quadrivalents do not exist. We hypothesized that this could be due to the smaller differences in map length between LGs in this study compared with that in sweetpotato [23]. Further studies using different mapping populations are needed to confirm the lack of correlation between proportions of quadrivalents.

As mentioned earlier, the error prior and the probability threshold for pairing configuration affect the proportion of quadrivalents. The larger the error prior, the lower the percentage of multivalency because erroneous recombinations are suppressed ((Fig. S5). Setting the probability threshold to 0.8 allowed only highly plausible pairing configurations to be included in the analysis. However, in the analysis using PolyOriginR (Table S2–S4), only an average of 78 individuals were included in the pairing configuration analysis for chr-1 of BM with the strict probability threshold of 0.8, whereas an average of 129 individuals were included for the other chromosomes. We hypothesized that the discrepancy in the number of individuals with reliable pairing configurations may have resulted in the exceptionally high quadrivalent proportion in chr-1 of BM. On the other hand, under a lenient probability threshold of 0.3, observed frequencies of bivalents and quadrivalents fluctuated significantly, as indicated by the p values of the χ^2^ test for chr-1 of SP ranging from 5.8 × 10^−6^ to 0.2797 (Tables S2–S13). Therefore, the probability threshold of 0.5 was considered suitable in this study.

In conclusion, we demonstrated that the newly developed sequencing method, named dpMIG-seq, was applicable to polyploids for the first time, providing beneficial information for optimizing genomic analysis in polyploids. By applying dpMIG-seq to the mapping population, the mode of inheritance in two tetraploid blueberry cultivars was revealed, including the probability of preferential pairing and varying double reduction rates along with different proportions of quadrivalents between chromosomes and cultivars. Our results not only strongly supported the findings of the latest study [25] but also significantly advanced the understanding of the differences in inheritance patterns between selections/cultivars. Our technical assessment of dpMIG-seq is expected to promote the application of this cost-effective and rapid genotyping technology in polyploid studies, thereby advancing genetic understandings within the challenging context of polyploids.

## Supplementary Material

Web_Material_uhae248

## Data Availability

Sequencing data were deposited in the Sequence Read Archive (SRA). The accession number for the resequencing data is DRA015015, that for the dpMIG-seq data is DRA016120, and that for the supplementary materials is DRA019089.
