# Supplementary material for: A low-cost dpMIG-seq method for elucidating complex inheritance in polysomic crops: a case study in tetraploid blueberry
Source: Hortic Res. 2024 Sep 4;11(11):uhae248. doi: 10.1093/hr/uhae248 (PMC11560368; doi:10.1093/hr/uhae248)
Supplement: Web_Material_uhae248 [file web_material_uhae248.zip › Supplementary_data_revise3.1.docx]

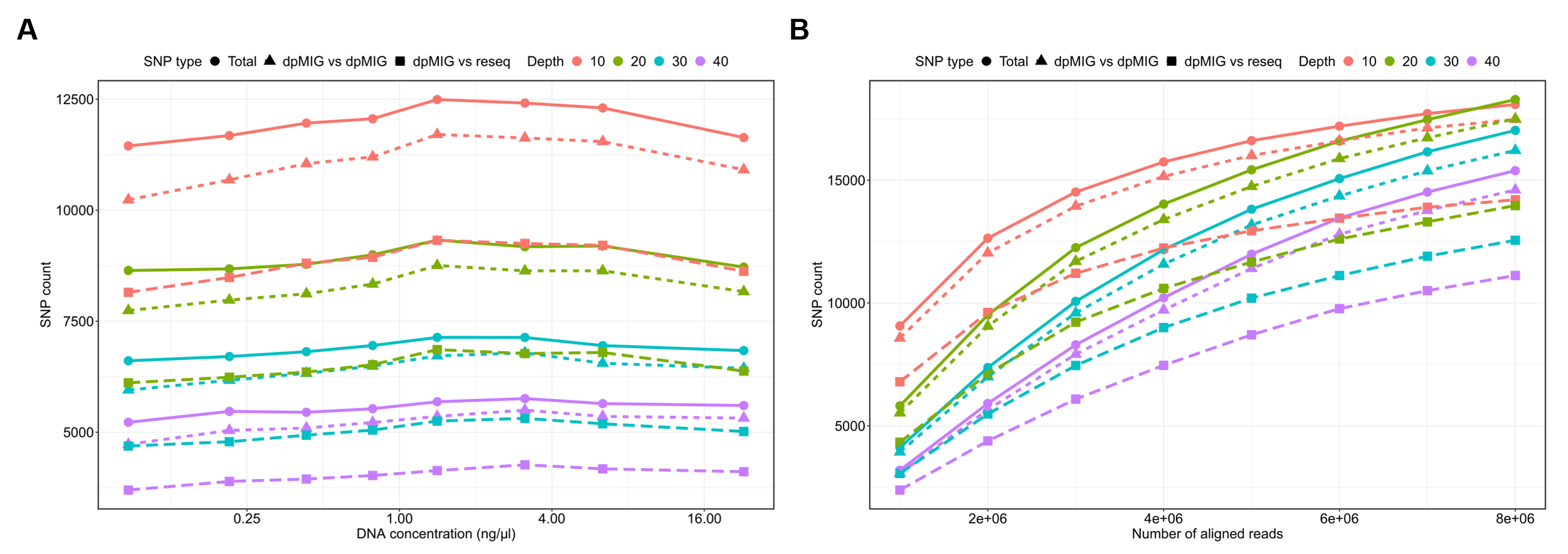


**Figure S1 The number of SNPs with concordant genotyping results depending on (A) DNA concentration and (B) the number of aligned reads**

These are the results of SNPs without missing allele dosage in both technical/biological replicates and resequencing data. The “Total” represents the total number of SNPs examined. The “dpMIG vs dpMIG” represents the number of SNPs that have concordant genotyping results between (A) technical replicates and (B) biological replicates. The “dpMIG vs reseq” represents the number of SNPs that have concordant genotype between dpMIG-seq and resequencing. For (A), the x-axis was log2-transformed.

**
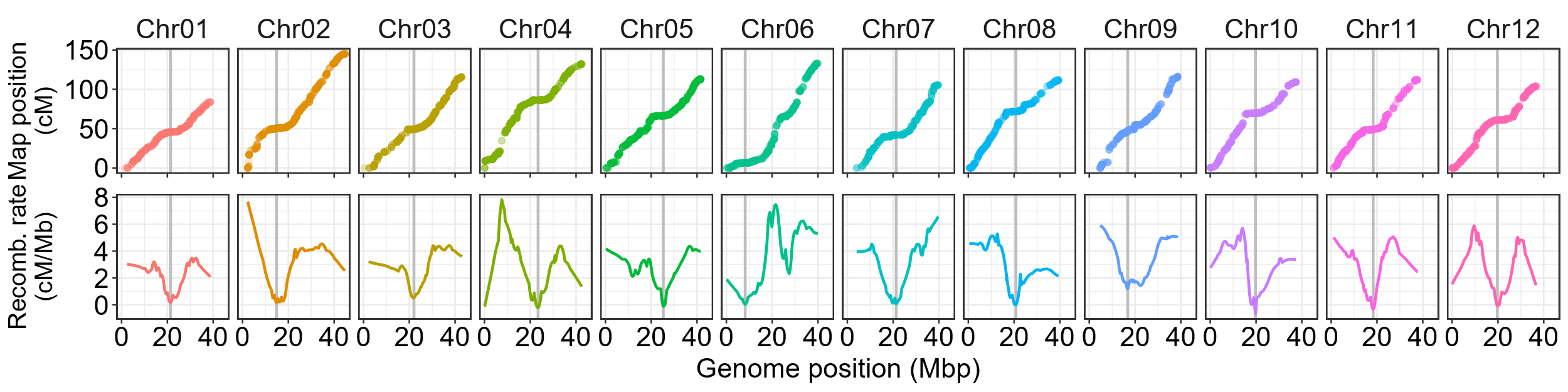
Figure S2 Collinearity between the linkage map and the longest 12 scaffolds of the ‘Draper’ genome assembly**

(Upper row) Marey maps depicting genetic distance plotted against physical position. (Lower row) Recombination rate across chromosomes. "Recomb. rate" on the y-axis denotes recombination rate. Gray vertical lines represent genomic regions with the lowest recombination rates.


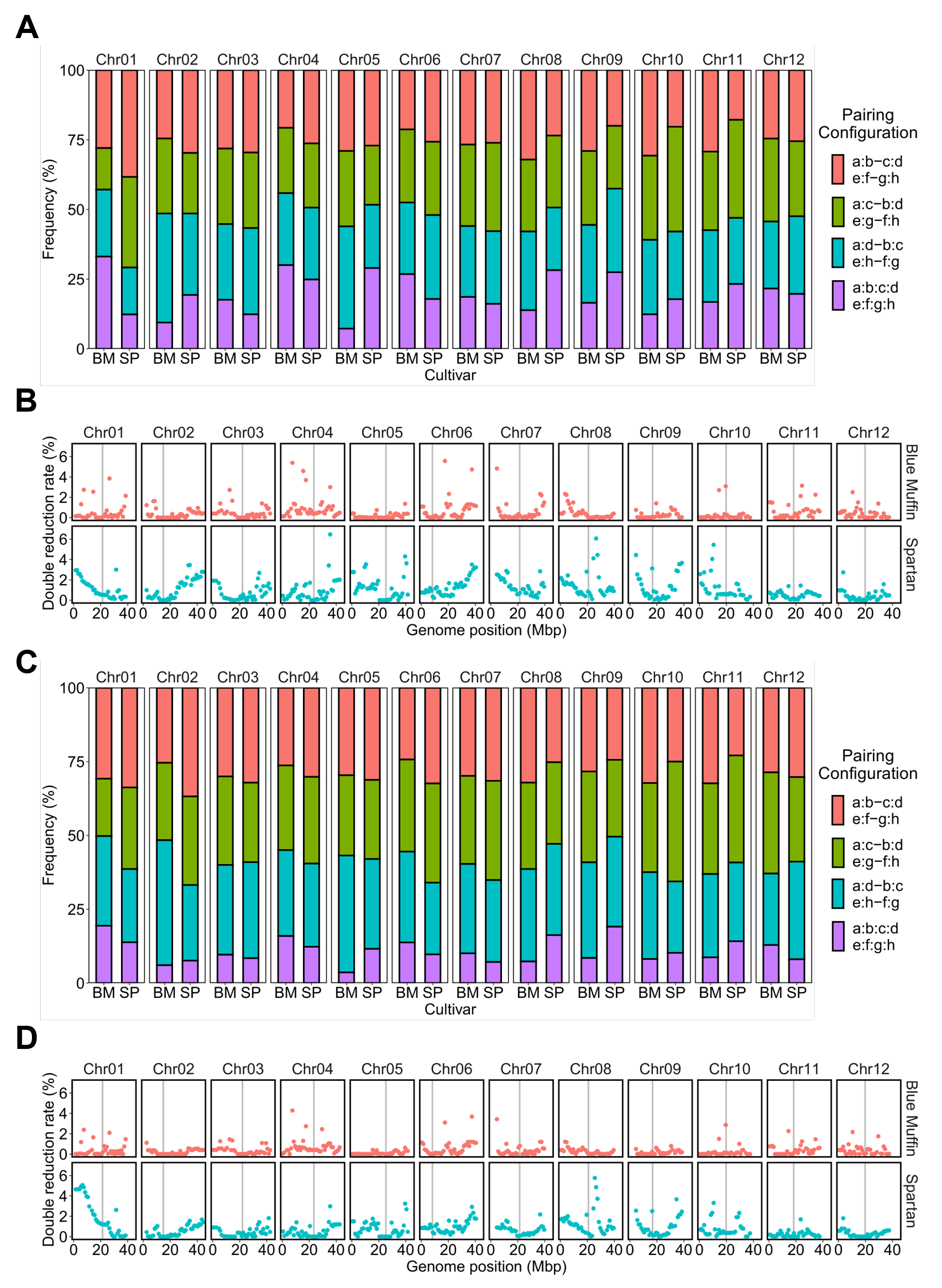


**Figure S3** **Double-reduction landscape when the error prior is 0.01**

Results estimated from the PolyOriginR and polyqtlR packages are shown in (A)-(B) and (C)-(D), respectively. (A), (C) Proportion of bivalents and quadrivalents when the probability threshold is 0.5. Paired chromosomes are indicated by “:”, e.g. a:b shows that homologous chromosome a and b form a bivalent. BM stands for ‘Blue Muffin’ and SP for ‘Spartan’. (B), (D) Double reduction rate across 12 linkage groups. Red and blue points represent ‘Blue Muffin’ and ‘Spartan’, respectively. Gray vertical lines represent genomic regions with the lowest recombination rates.


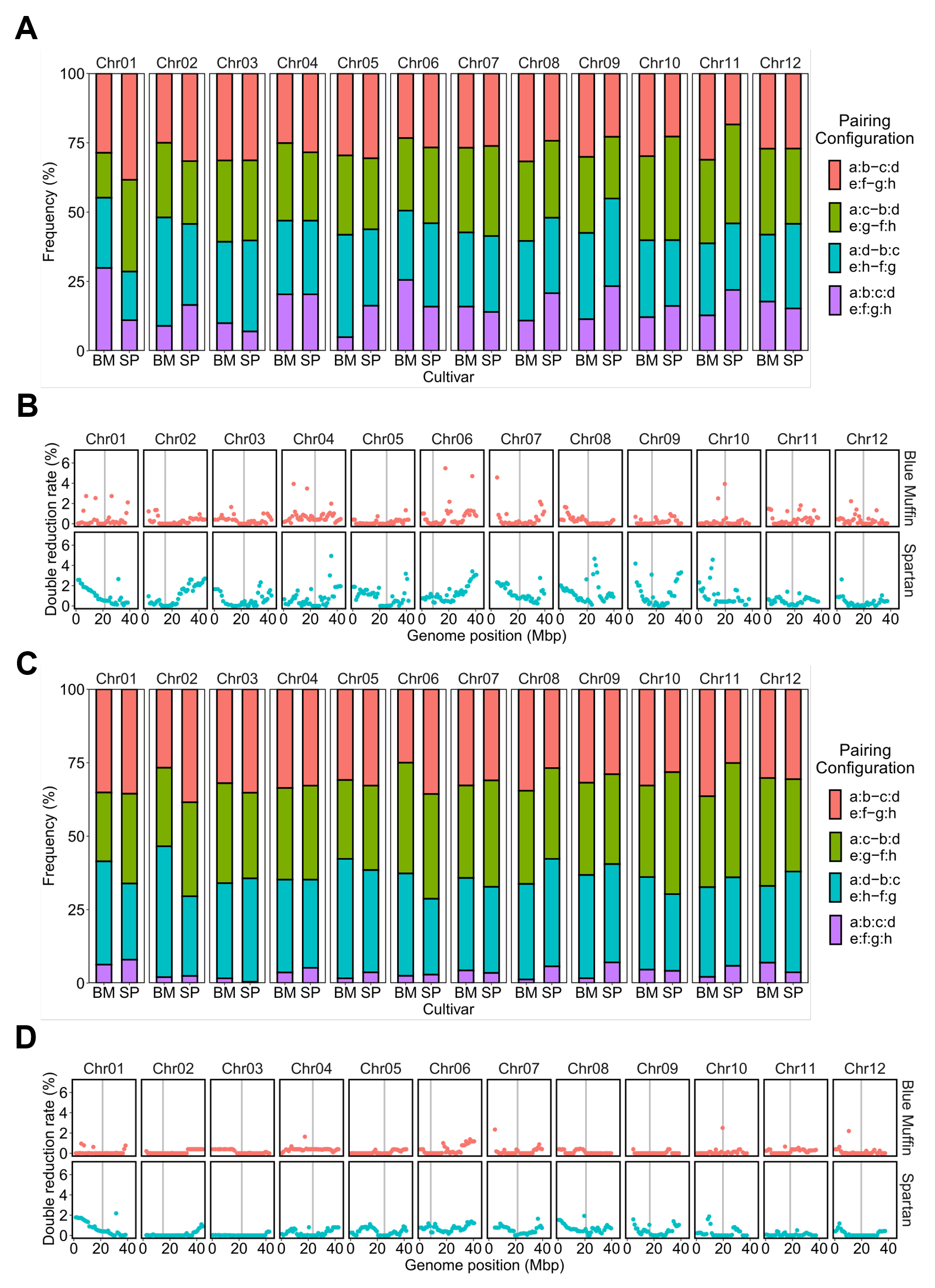


**Figure S4** **Double-reduction landscape when the error prior is 0.1**

Results estimated from the PolyOriginR and polyqtlR packages are shown in (A)-(B) and (C)-(D), respectively. (A), (C) Proportion of bivalents and quadrivalents when the probability threshold is 0.5. Paired chromosomes are indicated by “:”, e.g. a:b shows that homologous chromosome a and b form a bivalent. BM stands for ‘Blue Muffin’ and SP for ‘Spartan’. (B), (D) Double reduction rate across 12 linkage groups. Red and blue points represent ‘Blue Muffin’ and ‘Spartan’, respectively. Gray vertical lines represent genomic regions with the lowest recombination rates.


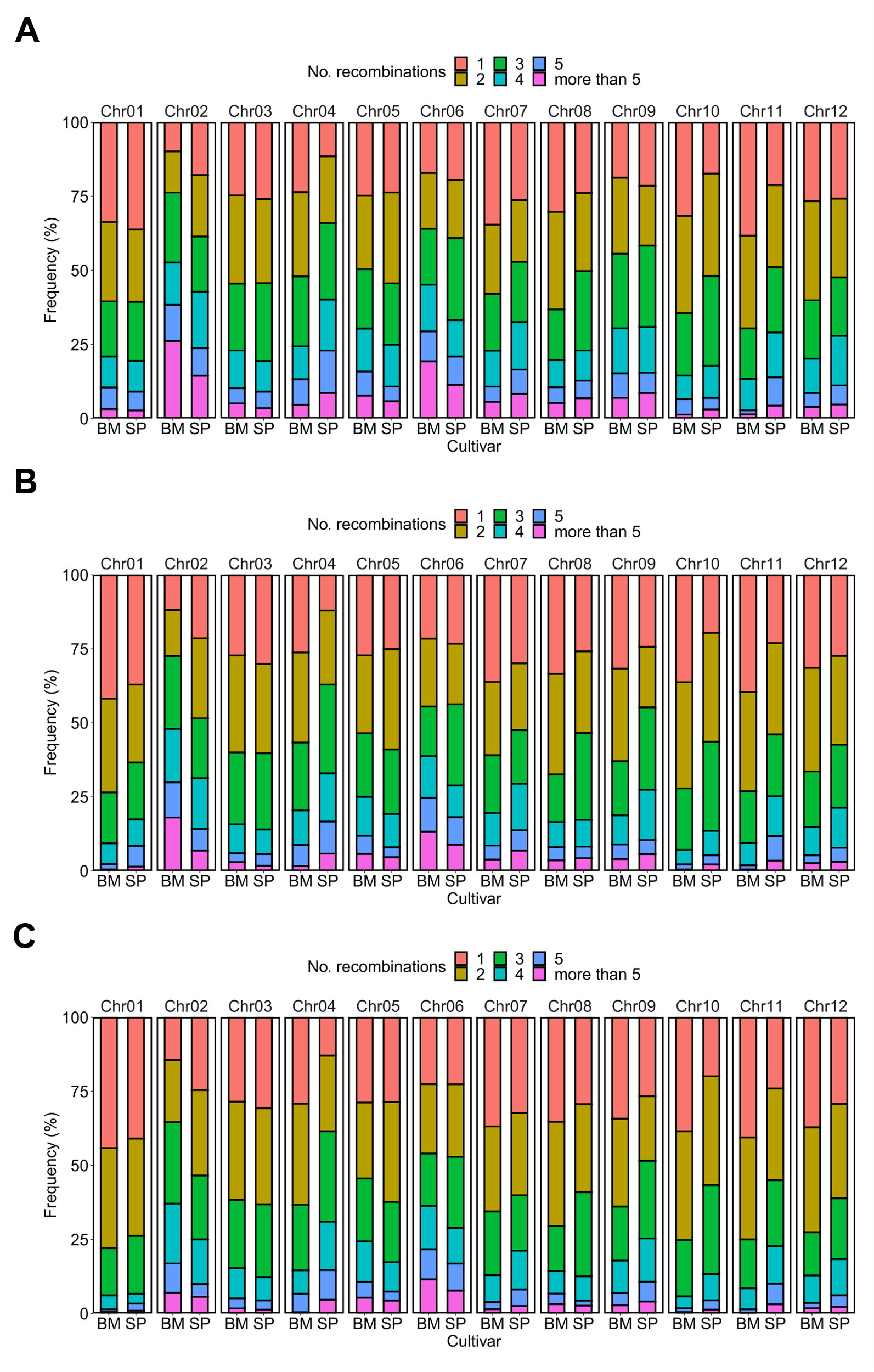


**Figure** **S5 Estimated number of recombinations across 12 linkage groups of ‘Blue Muffin’ and ‘Spartan’ when the probability threshold is 0.5**

(A), (B), and (C) are the results when the error prior is 0.01, 0.05, and 0.1, respectively. BM stands for ‘Blue Muffin’ and SP for ‘Spartan’.

**Table S1 First PCR primers for MIG-seq and dpMIG-seq used in this study**

These primers used for dpMIG-seq contain the degenerate oligonucleotide at the fourth and fifth bases from 3’ end. This table was prepared by summarizing a primer set named PS1 and PS1_4-5, which are listed in the Supporting Table of Nishimura et al. (2024).

**Reference**

Nishimura, K. et al. Degenerate oligonucleotide primer MIG-seq: an effective PCR-based method for high-throughput genotyping. Plant J. 118, 2296-2317 (2024).

**Table S2** **Observed frequencies of bivalents and quadrivalents estimated by the PolyOriginR package when the error prior is 0.01**

Probability denotes the threshold for plausible pairing configurations. Paired chromosomes are indicated by “:”, e.g. a:b shows that homologous chromosome a and b form a bivalent. χ^2^ test was performed to test for deviation from 1/3 for the observed frequencies of bivalents.

**Table S3 Observed frequencies of bivalents and quadrivalents estimated by the PolyOriginR package when the error prior is 0.05 and the probability threshold is 0.8 or 0.3**

Probability denotes the threshold for plausible pairing configurations. Paired chromosomes are indicated by “:”, e.g. a:b shows that homologous chromosome a and b form a bivalent. χ^2^ test was performed to test for deviation from 1/3 for the observed frequencies of bivalents.

**Table S4 Observed frequencies of bivalents and quadrivalents estimated by the PolyOriginR package when the error prior is 0.1**

Probability denotes the threshold for plausible pairing configurations. Paired chromosomes are indicated by “:”, e.g. a:b shows that homologous chromosome a and b form a bivalent. χ^2^ test was performed to test for deviation from 1/3 for the frequency of bivalent observations.

**Table S5 Observed frequencies of bivalents estimated by the PolyOriginR package when the error prior is 0.01**

Probability denotes the threshold for plausible pairing configurations. Paired chromosomes are indicated by “:”, e.g. a:b shows that homologous chromosome a and b form a bivalent. χ^2^ test was performed to test for deviation from 1/3. This result was obtained assuming only the formation of bivalent.

**Table S6 Observed frequencies of bivalents estimated by the PolyOriginR package when the error prior is 0.05**

Probability denotes the threshold for plausible pairing configurations. Paired chromosomes are indicated by “:”, e.g. a:b shows that homologous chromosome a and b form a bivalent. χ^2^ test was performed to test for deviation from 1/3. This result was obtained assuming only the formation of bivalent.

**Table S7 Observed frequencies of bivalents estimated by the PolyOriginR package when the error prior is 0.1**

Probability denotes the threshold for plausible pairing configurations. Paired chromosomes are indicated by “:”, e.g. a:b shows that homologous chromosome a and b form a bivalent. χ^2^ test was performed to test for deviation from 1/3. This result was obtained assuming only the formation of bivalent.

**Table S8 Observed frequencies of bivalents and quadrivalents by the polyqtlR package when the error prior is 0.01**

Probability denotes the threshold for plausible pairing configurations. Paired chromosomes are indicated by “:”, e.g. a:b shows that homologous chromosome a and b form a bivalent. χ^2^ test was performed to test for deviation from 1/3 for the frequency of bivalent observations.

**Table S9 Observed frequencies of bivalents and quadrivalents by the polyqtlR package when the error prior is 0.05 and the probability threshold is 0.8 or 0.3**

Probability denotes the threshold for plausible pairing configurations. Paired chromosomes are indicated by “:”, e.g. a:b shows that homologous chromosome a and b form a bivalent. χ^2^ test was performed to test for deviation from 1/3 for the frequency of bivalent observations.

**Table S10 Observed frequencies of bivalents and quadrivalents by the polyqtlR package when the error prior is 0.1**

Probability denotes the threshold for plausible pairing configurations. Paired chromosomes are indicated by “:”, e.g. a:b shows that homologous chromosome a and b form a bivalent. χ^2^ test was performed to test for deviation from 1/3 for the frequency of bivalent observations.

**Table S11 Observed frequencies of bivalents by the polyqtlR package when the error prior is 0.01**

Probability denotes the threshold for plausible pairing configurations. Paired chromosomes are indicated by “:”, e.g. a:b shows that homologous chromosome a and b form a bivalent. χ^2^ test was performed to test for deviation from 1/3. This result was obtained assuming only the formation of bivalent.

**Table S12 Observed frequencies of bivalents by the polyqtlR package when the error prior is 0.05**

Probability denotes the threshold for plausible pairing configurations. Paired chromosomes are indicated by “:”, e.g. a:b shows that homologous chromosome a and b form a bivalent. χ^2^ test was performed to test for deviation from 1/3. This result was obtained assuming only the formation of bivalent.

**Table S13 Observed frequencies of bivalents by the polyqtlR package when the error prior is 0.1**

Probability denotes the threshold for plausible pairing configurations. Paired chromosomes are indicated by “:”, e.g. a:b shows that homologous chromosome a and b form a bivalent. χ^2^ test was performed to test for deviation from 1/3. This result was obtained assuming only the formation of bivalent.

**Table S14 Comparison of approximate cost per sample for genotyping between dpMIG-seq, MIG-seq, and ddRAD-seq**

Items necessary for library construction described in Materials and Methods in this study were used for cost estimation of dpMIG-seq nad MIG-seq. ddRAD-seq followed Supplementary Materials and Methods for comparison. The term "No. SNPs" denotes the estimated number of SNPs from 5 million aligned reads (see Supplementary Materials and Methods for details).
