# Supplementary material for: A low-cost dpMIG-seq method for elucidating complex inheritance in polysomic crops: a case study in tetraploid blueberry
Source: Hortic Res. 2024 Sep 4;11(11):uhae248. doi: 10.1093/hr/uhae248 (PMC11560368; doi:10.1093/hr/uhae248)
Supplement: Web_Material_uhae248 [file web_material_uhae248.zip › Supplementary_m&m.docx]

**Supplementary Materials and Methods**

**Plant materials and DNA extraction**

Tetraploid southern highbush blueberry (*Vaccinium corymbosum* L. interspecific hybrids) ‘Blue Muffin’ was self-pollinated to create a selfing population. Seeds harvested from self-pollinated fruit were thawed and planted in pots in 2020. In 2021, total DNA was extracted from the parental cultivar and seven individuals from the selfing population using a DNeasy Plant Mini Kit (Qiagen, Hilden, Germany).

**MIG-seq and dpMIG-seq library construction, and sequencing**

Sequencing libraries for multiplexed inter-simple sequence repeat (ISSR) genotyping by sequencing (MIG-seq) ^[1, 2]^ and degenerate oligonucleotide MIG-seq (dpMIG-seq) ^[3]^ were constructed following the methods described in ^[3, 4]^ and “Sequencing and allele dosage estimation” section of the Materials and Methods in this study Initially, multiplex polymerase chain reaction (PCR) was performed using Multiplex PCR Assay Kit ver. 2 (TAKARA Bio Co. Ltd., Kusatsu, Japan) and primers (Table S1). Primers without degenerate oligonucleotide were used for MIG-seq, while primers with degenerate oligonucleotide at the fourth and fifth based from 3’ end were used for dpMIG-seq. The PCR conditions involved an initial denaturation step at 94°C for 1 min, followed by 25 cycles of denaturation at 94°C for 30 sec, annealing at 38°C for 1 min, extention at 72°C for 1 min, and a final extension at 72°C for 10 min. The resulting PCR product was diluted 50-fold to facilitate the second PCR step, which employed indexing primers ^[4]^ and PrimeSTAR GXL DNA Polymerase (TAKARA Bio Co. Ltd.). The second PCR conditions included an initial denaturation at 98°C for 30 sec, followed by 20 cycles of denaturation at 98°C for 10 sec, annealing at 54°C for 15 sec, extension at 68°C for 30 sec, and a final extension at 72°C for 10 min. Subsequently, the second PCR products were pooled, purified using AMPure XP (Beckman Coulter, Inc., CA, USA), and subjected to reconditioning PCR with conditions including at 98°C for 40 sec, 54°C for 15 sec, extension at 68°C for 30 sec, and a final extension at 72°C for 10 min. Following purification using AMPure XP, fragments of suitable length for sequencing were selected using SPRIselect (Beckman Coulter, Inc.).

**ddRAD-seq library construction and sequencing**

Sequencing library for ddRAD-seq was constructed following the methods described in Nishimura et al. (2024) ^[5]^, which was adapted from Peterson et al. (2012) ^[6]^ and Shirasawa et al. (2016) ^[7]^. Initially, two single-stranded adapters (40µL each) were mixed and subjected to heat denaturation at 98°C, followed by a controlled temperature decrease to 15°C to facilitate adapter annealing. Three enzyme combinations were employed: *Pst*I/*Eco*RI, *Eco*RI/*Hind*III, and *Pst*I/*Msp*I. Approximately 50 ng of DNA, quantified using a Nanodrop (Thermo Scientific, NanoDrop products, MA, USA), was digested with the respective restriction enzyme combinations at 37°C for 6 hours. The digested DNA was then mixed with the same volume of pre-annealed adapters. Adapter ligation was performed using the LigaFast^TM^ Rapid DNA Ligation System (Promega, WI, USA) with a DNA mixture: ligase: ligation buffer ratio of 2:3:5. Ligation was carried out at 23°C for 5 min, followed by inactivation at 70°C for 30 min. Indexing PCR conditions were: initial denaturation at 94°C for 3 min, followed by 25 cycles of denaturation at 98°C for 10 sec, annealing at 55°C for 30 sec, extensions at 68°C for 15 sec, and a final extension at 68°C for 5 min, using indexing primers described in Nishimura et al ^[5]^ and OKD plus Neo (Toyobo Co. Ltd., Osaka, Japan).

**Sequencing and SNP analysis**

The obtained libraries were sequenced on the Illumina HiSeq X platform using 151-cycle paired-end runs. Using fastp software (version 0.20.1) ^[8]^, raw reads were filtered with default settings except reads with a base-quality Phred score of less than 20 and a read length of less than 35, which were discarded. At the same time, for reads from the MIG-seq and pdMIG-seq libraries, 17 base primer sequences in the first PCR of MIG-seq and dpMIG-seq ^[3, 4]^ were trimmed. Clean reads were aligned to the 12 largest chromosomes of each homologous set from the ‘Draper’ reference genome ^[9]^ using BWA-MEM (version 0.7.17-r1188) ^[10]^. Single nucleotide polymorphism (SNP) calling was performed using the mpileup command in SAMtools program (version 1.13) ^[11]^ and the mpileup2snp command in VarScan (version 2.4.3) ^[12]^, and alignments with mapping quality less than 20 were discarded. SNPs were then filtered using VCFtools (0.1.16) ^[13]^ with the following criteria: (i) minor allele frequency of 0.05 (option --maf 0.05) and (ii) only biallelic loci. Depths of bases with a Phred score of 15 or greater were extracted per locus and per sample using vcfR (version 1.15.0) ^[14]^. To compare SNP counts between MIG-seq, dpMIG-seq, and ddRAD-seq, depths were divided by the number of aligned reads per library and per sample, and then multiplied by 5 million for adjustment. Subsequently, SNPs with a depth of 20 or more across more than half of the samples were counted.

**Supplementary References**

1. Suyama, Y. & Matsuki, Y. MIG-seq: an effective PCR-based method for genome-wide single-nucleotide polymorphism genotyping using the next-generation sequencing platform. Sci. Rep. 5, 16963 (2015).
2. Suyama, Y. et al. Complementary combination of multiplex high-throughput DNA sequencing for molecular phylogeny. Ecol. Res., 37, 171–181 (2022).
3. Nishimura, K. et al. Degenerate oligonucleotide primer MIG-seq: an effective PCR-based method for high-throughput genotyping. Plant J. 118, 2296–2317 (2024).
4. Nishimura, K. et al. MIG-seq is an effective method for high-throughput genotyping in wheat (*Triticum* spp.). DNA Res. 29, 1–13 (2022).
5. Nishimura, K. et al. Workflow for development of CAPS markers with one type of restriction enzyme to identify citrus cultivars. Tree Genet. Genomes (2024). in press.
6. Peterson, B.K., Weber, J.N., Kay, E.H., Fisher, H.S. & Hoekstra, H.E. Double digest RADseq: An inexpensive method for de novo SNP discovery and genotyping in model and non-model species. PLoS One 7, e37135 (2012).
7. Shirasawa, K., Hirakawa, H. & Isobe, S. Analytical workflow of double-digest restriction site-associated DNA sequencing based on empirical and *in silico* optimization in tomato. DNA Res. 23, 145–153 (2016).
8. Chen, S., Zhou, Y., Chen, Y. & Gu, J. fastp: an ultra-fast all-in-one FASTQ preprocessor. Bioinformatics 34, i884–i890 (2018).
9. Colle, M. et al. Haplotype-phased genome and evolution of phytonutrient pathways of tetraploid blueberry. GigaScience 8, 1–15 (2019).
10. Li, H. Aligning sequence reads, clone sequences and assembly contigs with BWA-MEM. arXiv preprint, [arXiv:1303.3997v2](https://arxiv.org/abs/1303.3997v2) (2013).
11. Li, H. et al. The sequence alignment/map format and SAMtools. Bioinformatics 25, 2078–2079 (2009).
12. Koboldt, D. et al. VarScan 2: Somatic mutation and copy number alteration discovery in cancer by exome sequencing. Genome Res. 22, 568–576 (2012).
13. Danecek, P. et al. [The Variant Call Format and VCFtools](http://dx.doi.org/10.1093/bioinformatics/btr330). Bioinformatics 27, 2156–2158 (2011).
14. Knaus, B.J. & Grünwald, N.J. VCFR: a package to manipulate and visualize variant call format data in R. Mol. Ecol. Resour. 17, 44–53 (2017).
